# Novel aspects on the pathogenesis of *Mycoplasma pneumoniae* pneumonia and therapeutic implications

**DOI:** 10.3389/fmicb.2014.00410

**Published:** 2014-08-11

**Authors:** Takeshi Saraya, Daisuke Kurai, Kazuhide Nakagaki, Yoshiko Sasaki, Shoichi Niwa, Hiroyuki Tsukagoshi, Hiroki Nunokawa, Kosuke Ohkuma, Naoki Tsujimoto, Susumu Hirao, Hiroo Wada, Haruyuki Ishii, Koh Nakata, Hirokazu Kimura, Kunihisa Kozawa, Hajime Takizawa, Hajime Goto

**Affiliations:** ^1^Department of Respiratory Medicine, Kyorin University School of MedicineMitaka, Japan; ^2^Department of Virology and Immunology, College of Veterinary Medicine, Nippon Veterinary and Animal Science UniversityMitaka, Japan; ^3^Gunma Prefectural Institute of Public Health and Environmental SciencesMaebashi, Japan; ^4^Bioscience Medical Research Center, Niigata University Medical and Dental HospitalNiigata, Japan; ^5^Infectious Disease Surveillance Center, National Institute of Infectious DiseasesTokyo, Japan

**Keywords:** *Mycoplasma pneumoniae* pneumonia, animal models, epidemiology, pathology, pathogenesis

## Abstract

*Mycoplasma pneumoniae* (Mp) is a leading cause of community acquired pneumonia. Knowledge regarding Mp pneumonia obtained from animal models or human subjects has been discussed in many different reports. Accumulated expertise concerning this critical issue has been hard to apply clinically, and potential problems may remain undiscovered. Therefore, our multidisciplinary team extensively reviewed the literature regarding Mp pneumonia, and compared findings from animal models with those from human subjects. In human beings, the characteristic pathological features of Mp pneumonia have been reported as alveolar infiltration with neutrophils and lymphocytes and lymphocyte/plasma cell infiltrates in the peri-bronchovascular area. Herein, we demonstrated the novel aspects of Mp pneumonia that the severity of the Mp pneumonia seemed to depend on the host innate immunity to the Mp, which might be accelerated by antecedent Mp exposure (re-exposure or latent respiratory infection) through up-regulation of Toll-like receptor 2 expression on bronchial epithelial cells and alveolar macrophages. The macrolides therapy might be beneficial for the patients with macrolide-resistant Mp pneumonia via not bacteriological but immunomodulative effects. This exhaustive review focuses on pathogenesis and extends to some therapeutic implications such as clarithromycin, and discusses the various diverse aspects of Mp pneumonia. It is our hope that this might lead to new insights into this common respiratory disease.

## Introduction

*Mycoplasma pneumoniae* (Mp) was first isolated in tissue culture from the sputum of a patient with primary atypical pneumonia by Eaton et al. (1944). This “Eaton's agent” was shown to be a *Mycoplasma* species in 1961. Chanock et al. succeeded in culturing Eaton's agent in mammalian cell-free medium and proposed the taxonomic designation Mp in 1963 (Chanock et al., 1962; Chanock, 1963). Mp is a unique organism that lacks a cell wall in any circumstances, and does not need a host cell for replication. This organism causes a variety of clinical presentations, from self-limiting to life-threatening. The disease severity seems to depend on the degree of host's defenses. In this review, we focused on the pathogenesis of Mp pneumonia from the perspective of host defenses, based on findings from our mouse models.

## Epidemiology

Mp is one of the most common pathogens of community-acquired pneumonia (CAP) in adults (Table 1). In general, both regional differences and varying periods of surveillance may influence the results of etiological studies of infectious diseases. Table 1 summarizes the proportions of adult Mp pneumonia among CAP populations enrolled in several large-scale studies conducted in various countries (Marston et al., 1997; Ngeow et al., 2005; Arnold et al., 2007; Von Baum et al., 2009; Cilloniz et al., 2011). Mp pneumonia accounted for 10.6–17.0 and 3.0–20.8% of CAP in out- or in-patients settings, respectively, and the frequency of ICU admission was relatively low (2–3.6%). Arnold et al. showed that Mp is the most common atypical pneumonia pathogen, accounting for 11–15% of CAP throughout the world (Arnold et al., 2007). Serological studies in Denmark over a 50-year period showed that Mp infections exhibit epidemic periodicity every 3–5 years, but this trend now seems to be getting obscured (Lind et al., 1997). Mp pneumonia occurs at any age, but the incidence is less common in elderly, as compared with young, adults (Lim et al., 2009), and is highest among school-aged children (Foy et al., 1979).

**Table 1:** **Prevalence of *Mycoplasma pneumoniae* pneumonia in CAP**.

**Author**	**Country**	**Year**	***N***	**Out-Pts%**	**Ward%**	**ICU%**	**Total%**	**Mortality%**
Cilloniz	Spain	1996–2008	1463	17	3	2	4	3.1
Ngeow	Asia	2001–2002	926	ND	ND	3.6	11.4	ND
Baum	German	2002–2006	4532	10.6	4.7		6.8	0.7
Marston	USA	1991	1938	ND	20.8^**^ (5.4^*^)		20.8^*^ (5.4^*^)	ND
Arnold	Whole world	2001–2006	4337	ND	12		12	ND

(Marston et al., 1997; Ngeow et al., 2005; Arnold et al., 2007; Von Baum et al., 2009; Cilloniz et al., 2011).

* definite case.

** definitive and possible cases.

N, number; ND, not determined; Out-Pts, out patients.

Macrolides were recommended for treatment of microbiologically defined Mp pneumonia. However, macrolide-resistant Mp was isolated from Japanese children, and the incidence was increasing in the early 2000s (Matsuoka et al., 2004). There was a major concern that macrolide-resistant Mp had increased locally and was spreading throughout the world. In East Asia, macrolide-resistant Mp rapidly increased and became the cause of the majority of clinically-proven Mp in both children and adults. The prevalence of macrolide-resistant Mp varies among countries and age groups (Averbuch et al., 2011; Akaike et al., 2012; Miyashita et al., 2012; Spuesens et al., 2012; Uldum et al., 2012; Yamada et al., 2012; Yoo et al., 2012; Dumke et al., 2013; Eshaghi et al., 2013; Pereyre et al., 2013; Wu et al., 2013; Zhao et al., 2013) (Table 2). For example, over 90% of isolated Mp in China was macrolide resistant, while no macrolide-resistant Mp was found in the Netherlands. Generally, it became highly prevalent in East Asian countries including China, Japan and South Korea, while being a medium or low prevalent in North America and Europe, respectively. Macrolide-resistant Mp is reportedly more prevalent in children, and the predominant point mutation found was A2063G in domain V of 23S rRNA. Aside from geographical and racial differences between individual studies, the application of different diagnostic techniques or criteria might affect the epidemiology of Mp pneumonia in each study.

**Table 2:**
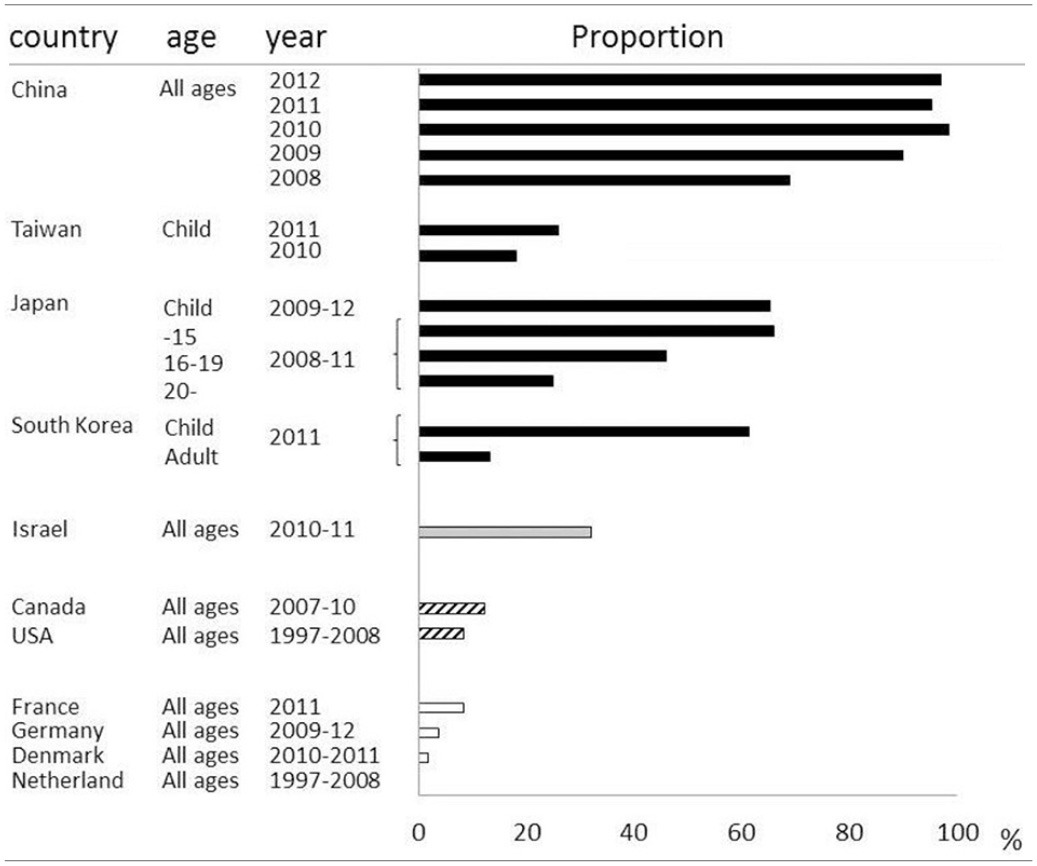
**Proportions of macrolide-resistant *Mycoplasma pneumonia***.

## Human pathology and bronchoalveolar lavage fluid

### Pathology

Studies focused on the pathological description of human Mp pneumonia have rarely been reported. However, pathological examinations have been conducted on several different types of specimens that were sampled using different techniques; e.g., autopsy specimens (Parker et al., 1947; Maisel et al., 1967; Benisch et al., 1972; Meyers and Hirschman, 1972; Halal et al., 1977; Kaufman et al., 1980; Koletsky and Weinstein, 1980), open lung biopsy specimens (Coultas et al., 1986; Rollins et al., 1986; Llibre et al., 1997; Ebnother et al., 2001; Wachowski et al., 2003), video-assisted thoracic surgery (VATS) specimens (Chan et al., 1999) and transbronchial lung biopsy specimens (Ganick et al., 1980; Nakajima et al., 1996; Ohmichi et al., 1998). According to these reports, the most characteristic pathological feature of human Mp pneumonia is a marked plasma cell-rich lymphocytic infiltration in the peri-bronchovascular areas (PBVAs), with accumulations of macrophages, neutrophils, and lymphocytes in the alveolar spaces (Parker et al., 1947; Coultas et al., 1986; Rollins et al., 1986). The presence of plasma cells in PBVAs might reflect up-regulation of humoral immunity via Mp infection.

### Bronchoalveolar lavage fluid (BALF) findings

There have been several case series focused on BALF obtained from human Mp pneumonia patients (Hayashi et al., 1986, 1993, 1998; Yano et al., 2001); those studies demonstrated varying levels of monocytes, polymorphonuclear leukocytes (PMNs), lymphocytes, eosinophils, and total cell counts. Among them, PMNs and lymphocytes counts were relatively more increased than the other cell types. The CD4 to CD8 ratios in the BALF were also elevated, and ranged from 2.1 (Hayashi et al., 1986) to 3.5 ± 2.1 (Hayashi et al., 1993), irrespective of the sampling timing.

## Pathogenesis

### Animal models

The incidence of Mp pneumonia is relatively low among the elderly over 70 years old or children less than 5 years old. This led to the hypothesis that elderly persons must be repeatedly exposed to and respond immunologically to the organism with clinical or subclinical progression. Indeed, as for cellular immunity, Brunner et al. have suggested that the occurrence of clinical disease in adults is favored by prior sensitization induced by infection at an early age, causing large or small mononuclear cell reactions (Brunner et al., 1973). This cellular response, lasting several years, could be proved by Mp antigen-induced lymphocyte transformation of cell suspensions from previously infected patients (Biberfeld et al., 1974; Biberfeld, 1974). It is important for us to understand immune responses attributed to Mp pneumonia.

**Figure 1:**
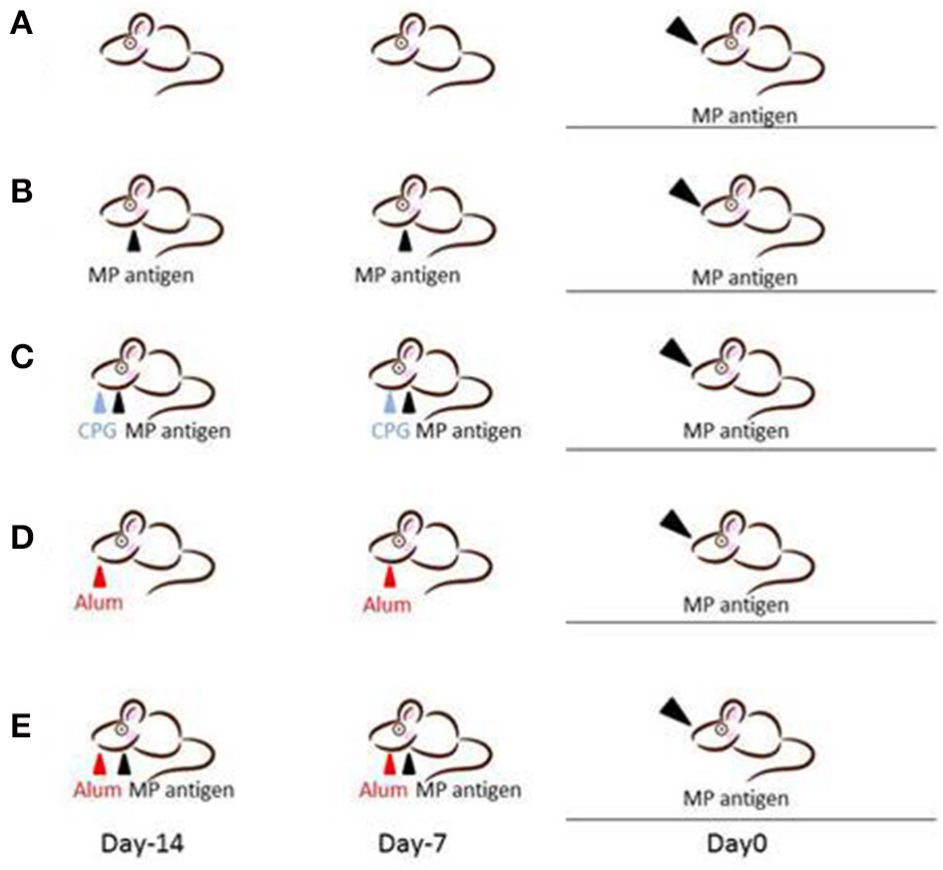
**Female BALB/c (7-week-old) mice were inoculated intratracheally with Mp antigen with or without pre-immunization**. Pre-immunization was carried out by intraperitoneal injection at 7 and 14 days prior to the intratracheal (IT) challenge. Model A: IT without pre-immunization. Model B: IT after twice pre-immunizing with Mp antigen alone. Model C: IT after twice pre-immunizing with Mp antigen plus CpG. Model D: IT after twice pre-immunizing with alum alone. Model E: IT after twice pre-immunizing with Mp antigen plus alum. One week following the last immunization, mice underwent IT with 50 μg of Mp antigen. Bronchoalveolar lavage fluid (BALF) and lung specimens were obtained before this process and 8, 24, 48, 96, and 168 h after IT. Mp: mycoplasma.

**Figure 2:**
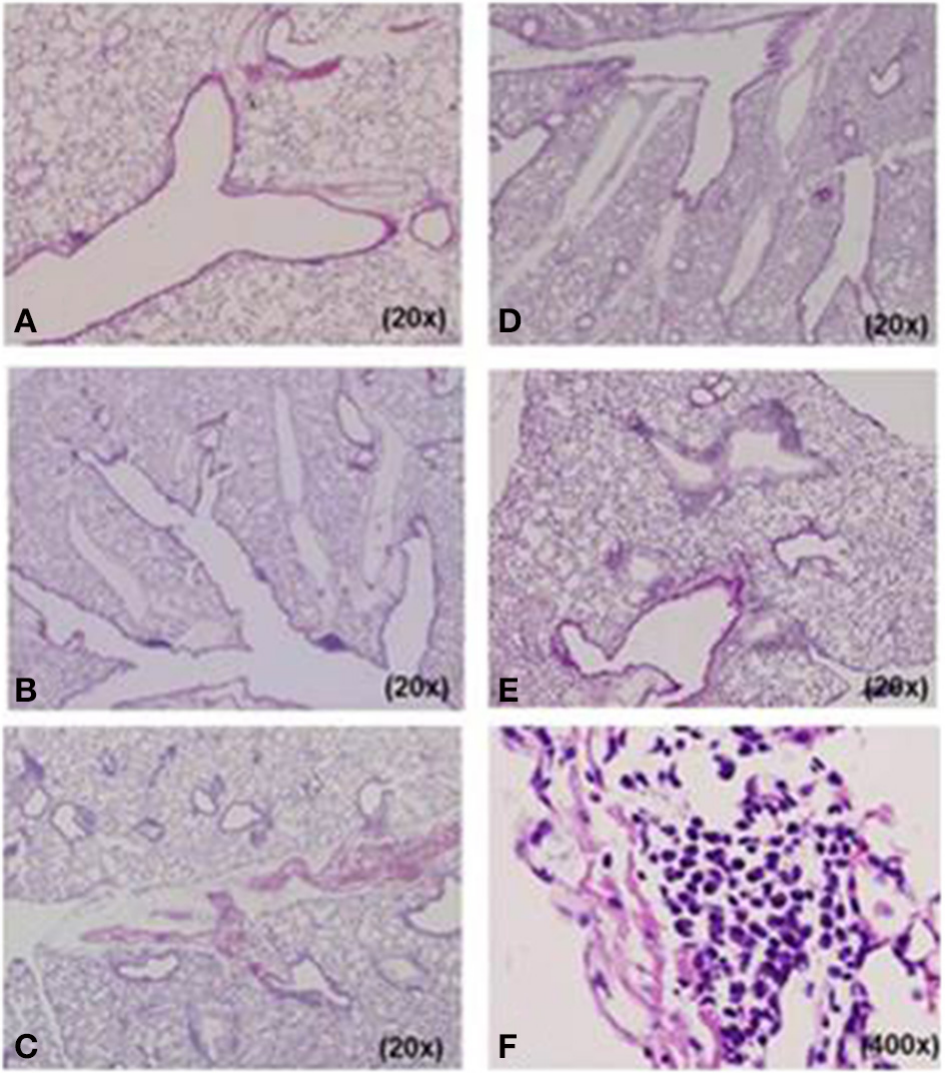
**Histopathological examination of lung specimens**. At 96 h post-intratracheal (IT) challenge, no inflammatory cells were seen in model **(A)** A or **(B)** B specimens. Mild to moderate lymphocyte infiltration was observed in the peribronchovascular area (PBVA) of **(D)** model D tissue. Models **(C)** C and **(E)** E had more severe lymphocyte infiltration in the PBVA. Plasmacyte infiltration within the PBVA was only recognized in models D and E, and the number of infiltrating plasmacytes was significantly higher in model E than model D. **(F)** High power magnification of E. Hemotoxylin and eosin stain. **A–F**, 200x; **E**, 400x.

We designed five different mouse models for Mp pneumonia (Figure 1) to examine the resulting pathology in animals having various immune status (Saraya et al., 2007b, 2011; Saraya, 2013). Animals were peritoneally immunized with various regimens (one per model) once a week (on days −14 and −7), then 1 week after the last immunization the animals were intratracheally (IT) challenged with sonicated Mp antigen, as previously reported (Saraya et al., 2011). Among those models, only groups immunized with Mp antigen and alum adjuvant (Figure 1E) or CpG (Figure 1C) developed severe lymphocytic infiltration into PBVAs at 96 h after IT (Figures 2C,E) while, no inflammatory cells were seen on models A and B (Figures 2A,B). However, the pathognomonic feature for human Mp pneumonia was reconstructed only in models D and E, in which lymphoplasmacytic infiltration into PBVAs occurred 96 h post-IT (Figures 2D,E). Those results suggest that enhanced host immune responses, as occurred in models C and E, against Mp antigen are required for persistent inflammation in the lung, as well as Th2 characteristics (produced by use of Th2 adjuvant, as in models D and E) causing plasma cell infiltration into the PBVAs, but not Th1 characteristics (produced by use of the Th1 adjuvant, CPG, as in the model depicted in Figure 1C). Aluminum hydroxide adjuvant, named alum, is well-known for initiating strong antigen-specific Th2 responses in the absence of interleukin(IL)-4- or IL-13-mediated signaling (Brewer et al., 1999); Th2 predominant characteristics might be required to generate typical Mp pneumonia, even in humans. Previous studies showed that the histopathological score of Mp pneumonia is significantly higher in infected BALB/c mice (Th2 predominant) than in C57BL/6 mice (Th1 predominant) through the late phase, suggesting differences in host reactions against intranasally-inoculated live Mp (Fonseca-Aten et al., 2005). Tanaka et al. (1996) describe the different pathological findings in an *M. pulmonis*–infected mouse model for treatment with IL-2 (Th1 up-regulated) vs. cyclosporine A (Th1 down-regulated).

Thus, the severity of Mp pneumonia seems to depend on the host immune response to the infection through a complexity of various mechanisms, including an allergic reaction to Mp, Mp virulence, host defenses, and polarization toward Th1 or Th2 predominance, to name a few. In the context of allergic reaction, IgE antibodies specific to Mp were detected in serum samples from patients with Mp pneumonia, suggestive of IgE-mediated hypersensitivity (Tipirneni et al., 1980; Yano et al., 1994; Seggev et al., 1996) as well as an involvement in asthma attacks (Henderson et al., 1979; Biscardi et al., 2004). In this review, we will further discuss the pathomechanisms of Mp pneumonia from the perspective of the virulence of Mp and presumed host defenses based on findings obtained from our experimental mouse models.

### Virulence of Mp

#### Lipoproteins

Lipoproteins from various Mycoplasma species have potent inflammatory properties. Three lipoproteins/lipopeptides of *M. fermentans* origin, macrophage-activating lipopeptide-2 (MALP-2), P48, and M161Ag (identical to MALP-404), reportedly modulate the host immune system via Toll-like receptor (TLR)-2/TLR-6 signaling (Takeuchi et al., 2000; Luhrmann et al., 2002; Seya and Matsumoto, 2002). Genes for more than 30 different Mp lipoproteins have been reported (Himmelreich et al., 1997). Shimizu et al. reported that the mycoplasma-derived lipoproteins N-ALP1/N-ALP2 (Shimizu et al., 2008) and F_0_F_1_-ATPase (Shimizu et al., 2005) activated NF-kβ via TLR-1, 2 or TLR-1, 2, 6 signaling, respectively. Stimulation of these TLRs has been known to be related to production of chemokines (Brant and Fabisiak, 2008; Andrews et al., 2013) that promote lymphocyte and neutrophil trafficking and inflammation in the lung.

#### CARDS (Community Acquired Respiratory Distress Syndrome) toxin

Kannan et al. first demonstrated the possibility that Mp produces the CARDS toxin that is involved in the mediation of disease (Kannan et al., 2005). The CARDS toxin is an ADP-ribosylating and vacuolating toxin, with homology to the S1 subunit of pertussis toxin, that has a high affinity for surfactant protein-A, suggesting a physiological role for the toxin in the pulmonary compartment. In mice, intranasal inoculation of recombinant CARDS toxin caused an increased level of pro-inflammatory cytokines IL-1α, 1β, 6, 12, 17, Tumor necrosis factor(TNF)-α, and Interferon-gamma (IFN)-γ together with elevation of Keratinocyte chemoattractant(KC), IL-8, regulated on activation, normal T cell expressed and secreted (RANTES), and G-CSF (Hardy et al., 2009). However, to our knowledge, there have been no reports of CARDS toxin identified in human respiratory specimens.

#### Other factors

Mp produces a soluble hemolysin (Somerson et al., 1963, 1965), hydrogen peroxide and superoxide radicals, which produce oxidative stress in the respiratory epithelium, resulting in both structural and functional deterioration of cilia (Waites and Talkington, 2004). Stimulation of human respiratory epithelial cells (A549 cells) *in vitro* with Mp lysate (MPL) induced IL-8 production (Sohn et al., 2005). MPL induced IL-8 release in a time- and dose-dependent manner together with activation of extracellular signal-regulated kinase (ERK), which was inhibited by PD98059, a specific inhibitor of ERK. Chmura et al. (2003) reported that the Mp membrane fraction induced IL-8 on BEAS-2B human bronchial epithelial cells. Our report (Hirao et al., 2011) also demonstrated activation of mitogen-activated protein kinase (MAPKs) on the alveolar macrophage-like cell line, RAW264.6, by stimulation with Mp antigen, as confirmed by significant suppression of IL-6 and TNF-α production after preceding treatment with an MAPKs inhibitor such as parthenolide (PAR: NF-kB inhibitor), SB20580 (SB, p38-linked signal of inhibitor), or LY294002 (LY, PI-3K inhibitor). Thus, Mp antigen or live Mp can induce inflammatory cytokines in bronchial epithelial cells and in alveolar macrophages (AMs).

### Host defenses

#### Cellular immunity

Biberfeld et al. reported that the peripheral lymphocyte response to a sonicate of Mp organisms or a membrane fraction was significantly higher in recently infected patients than in healthy patients (Biberfeld et al., 1974). The positive responsiveness to sonicated Mp antigen was demonstrable up to 10 years after infection. Others also reported on the *in vitro* response of human peripheral lymphocytes to Mp antigen (Fernald, 1972; Biberfeld, 1974), while tuberculin anergy in patients with Mp pneumonia was noted soon or fairly soon after onset. This has been speculatively explained by the possibility that (1) lymphocytes and macrophages needed for the skin reaction to tuberculin are engaged in the immune response to the infecting agent, or (2) a transient change of the T lymphocyte population occurs (Biberfeld and Sterner, 1976). Tanaka et al. reported that the rate of positive tuberculin tests during the acute stage of Mp pneumonia was higher in patients with the nodular type of pulmonary lesions on thoracic computed tomography than those having the consolidation pattern. This finding suggests that the level of current cell-mediated immunity might influence the pattern of pulmonary lesions. Another study showed that delayed hypersensitivity was noted on skin testing with Mp antigen of patients with Mp pneumonia (Mizutani et al., 1971).

However, to our knowledge, no direct evidence from patients with Mp pneumonia has been reported regarding the reactivity of BALF lymphocytes to Mp antigen. In other words, it is still under debate whether the lung inflammation of Mp pneumonia is a specific reaction to the Mp antigen.

**Figure 3:**
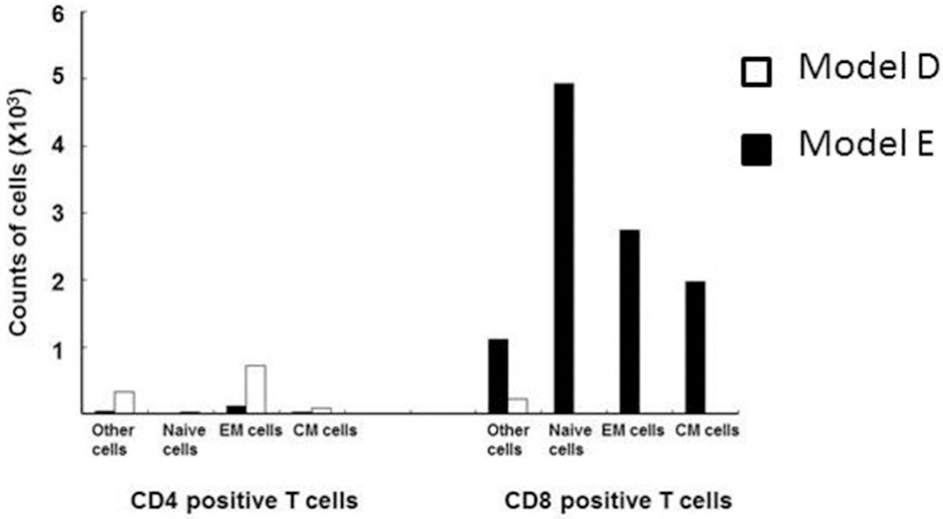
**Flow cytometry analyses of CD3 positive lymphocytes from the BALF of models D (□) and E (■) at 96 h post-intratracheal challenge**. CD8-positive cells predominated, and consisted of naïve cells (CD62L+^hi^/ CD44+^lo^), effector memory cells (CD62L+^lo^/CD44+^hi^), and central memory cells (CD62L+^hi^/CD44+^hi^), in descending order.

In consideration of this question, Saraya et al. (2011) demonstrated a lack of specific response of lymphocytes in the BALF to Mp antigen 96 h post-IT using the 3H-thymidine uptake test in an Mp pneumonia mouse model (Figures 1D,E). The BALF cells in the lymphocyte gate were 35.8% CD3 positive and 57.6% CD3 negative. Among the CD3 positive cells, CD4−/CD8− cells were predominant. The CD4 to CD8 ratio was 0.02, which was a lower value than that of human Mp pneumonia patients (Hayashi et al., 1986, 1993), and the CD8 positive cells consisted of naïve cells (CD62L+^hi^/ CD44+^lo^), effector memory cells (CD62L+^lo^/CD44+^hi^), and central memory cells (CD62L+^hi^/CD44+^hi^), in that order (Saraya et al., 2007a) (Figure 3). Cellular immunity seemed to play an important role in development of Mp pneumonia (Foy et al., 1973; Broughton, 1986); the results given above might indicate that non-specific reactions to Mp antigen govern the severity of lung inflammation.

#### Humoral immunity

The humoral immune responses in Mp pneumonia were elucidated by the discovery of autoimmune-mediated phenomena involving cross-reactive antibodies to host organs. Neurologic manifestations following Mp infection can occur as a result of molecular mimicry by carbohydrate moieties of the abundant glycolipids in the Mp membrane and the lipoglycan capsule (Ang et al., 2002; Yuki, 2007). Autoimmune hematologic disorders can also occur following Mp infection—transient brisk hemolytic anemia, termed “paroxysmal cold hemogloblinuria.” As for lung inflammation, how humoral immunity contributed to Mp pneumonia was unknown. However, patients with humoral deficiency seemed to become chronic carriers of Mp (Taylor-Robinson et al., 1980) or to undergo repeated episodes of Mp pneumonia (Roifman et al., 1986) or severe arthritis (Taylor-Robinson et al., 1978; Johnston et al., 1983), phenomena indicating that humoral immunity plays a role in protection against these organisms.

#### Cytokine profile in blood and BALF

Cytokines are important components of the lung defense mechanism and inflammation (Yang et al., 2004). Here we describe findings obtained from human patients and mouse models of Mp pneumonia.

***Cytokines in BALF of human Mp pneumonia***. A few studies have been reported concerning cytokine profiles in the BALF of human Mp pneumonia patients. Koh et al. reported that IL-4 levels and IL-4/IFN-γ ratios in BALF are significantly higher in children with Mp pneumonia than in patients with pneumococcal pneumonia or control participants (Koh et al., 2001). This suggests that a Th2-like cytokine response in Mp pneumonia is predominant, representing a favorable condition for IgE production. Yano et al. described an increased level of eosinophil cationic protein in BALF of all 10 Mp pneumonia patients studied, supporting the allergic aspects of Mp pneumonia (Yano et al., 2001).

**Figure 4:**
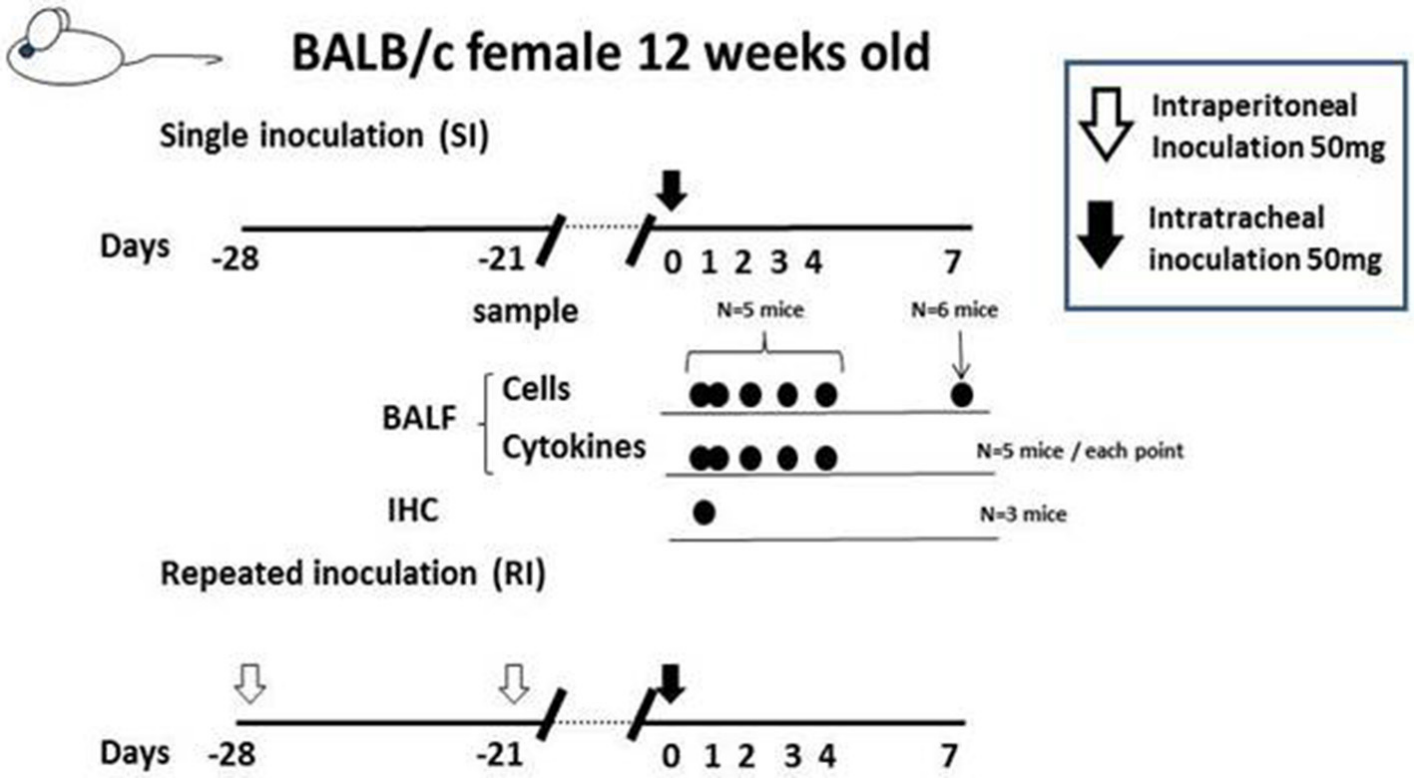
**The “no adjuvant used” model was created by intraperitoneally immunizing with only Mp antigen twice, on days −28 and −21 (for the RI, repeated inoculation, group) or without pre-treatment (for the SI, single inoculation, group), followed by intratracheal challenge with Mp antigen at day 0 for both groups**. Those two models were evaluated at days 0, 1 2, 3, 4, and 7 by examination of bronchoalveolar lavage fluid or lung pathology.

***Cytokine profile of BALF in Mp pneumonia mouse models***. Previous reports of mice inoculated with live Mp described that Mp induced an increase in BALF of the concentrations of IL-17, KC, TNF-α, IL-6, IFN-γ, and IL-12 (Fonseca-Aten et al., 2005; Chu et al., 2006; Salvatore et al., 2007, 2008; Wu et al., 2007). Likewise, we demonstrated that our model E (Figure 1) mice had a significant increase in the levels of BALF cytokines, including IL-6, MCP-1, and RANTES, 24 h post-IT, when compared to those of model D mice (Figure 1) (Saraya et al., 2011), which was thought to be attributable to antecedent immunization with Mp antigen. Regarding the allergic aspect, Mp infection in airway epithelial cells can contribute to the pathogenesis of chronic asthma by inducing RANTES and tumor growth factor-β1 (Sohn et al., 2005). We also generated another mouse model (in which no adjuvant was used), as reported by Kurai et al. (2013a), in which mice were intraperitoneally immunized with only Mp antigen twice, on day −28 and day −21 (RI, repeated inoculation, group) or had no pretreatment (SI, single inoculation, group), followed by IT challenge with Mp antigen on day 0 for both groups (Figure 4). In this RI model, the levels of proinflammatory or Th2 cytokines in BALF, including IL-17, KC, IL-6, TNFα, and IL-4, were significantly higher than those of the SI model mice. Furthermore, immunohistochemical analysis of lung tissues collected on day 1 revealed IL-23 positive alveolar macrophages together with elevation of IL-17 both in the BALF and in the supernatants of lung-derived cells cultured with Mp antigen, which suggested activation of the IL-23/IL-17 axis (Iwakura and Ishigame, 2006). Likewise, Wu et al. reported that Mp infection of mouse lungs can be prolonged when IL-23 mediated IL-17 production is neutralized (Wu et al., 2007).

***Cytokine profile of blood in human Mp pneumonia***. Tanaka et al. reported that serum levels of IL-18 were elevated during the acute phase of Mp pneumonia (Tanaka et al., 2002), which suggested IL-18 and Th1 cytokines may play a significant role in the immunopathologic responses in Mp pneumonia. Conversely, other reports described polarization to Th2 in Mp pneumonia, because of increased levels of eosinophil cationic protein (63%, 17 of 27 cases) (Yano et al., 2001) or the detection of IgE antibody specific for Mp (Tipirneni et al., 1980; Yano et al., 1994; Seggev et al., 1996), indicating an allergic aspect of human Mp pneumonia. Esposito et al. reported that children with acute Mp infection and wheeze had higher IL-5 concentrations than did healthy controls (Esposito et al., 2002). Matsuda et al. reported that serum IFN-γ, IL-6, and IP-10 (Interferon γ-induced protein 10) levels were higher in patients infected with macrolide-resistant Mp genotypes than were those in patients infected with conventional Mp strains (Matsuda et al., 2013).

#### What are the key players leading to lung inflammation in Mp pneumonia?

**Figure 5:**
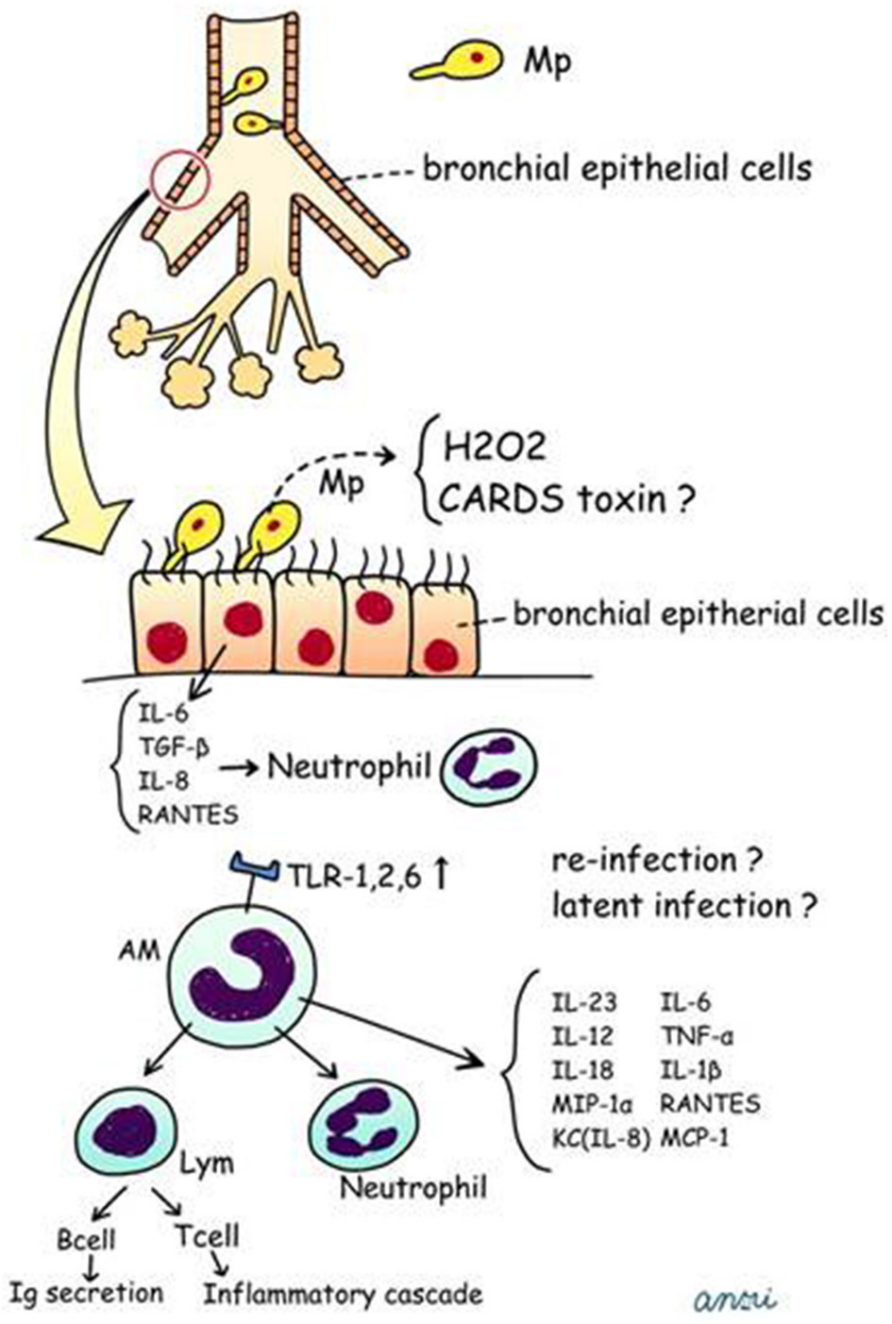
**Postulated schema for pathogenesis of human Mp pneumonia**. CARDS, Community Acquired Respiratory Distress Syndrome; TNF, tumor necrosis factor; RANTES, regulated on activation; normal T cell expressed and secreted; MCP-1, monocyte chemotactic protein-1.

We have postulated a process for the generation of human Mp pneumonia, which is described in Figure 5 and in the following sections.

***Bronchial epithelial cells***. Mp attaches to ciliated respiratory epithelial cells at the base of the cilia by means of a complex terminal organelle at one end of the elongated organism, which is mediated by interactive adhesins and accessory proteins clustered at the tip of the organelle. Briefly, Mp attaches to the bronchial epithelial cells via P1 adhesin (Razin and Jacobs, 1992), P30, and other structures (HMW1, HMW2, HMW4, HMW5, P90, and P65) (Waites and Talkington, 2004). Mp produces hydrogen peroxide and superoxide radicals, which induce oxidative stress in the respiratory epithelium. Dakhama et al. reported that Mp upregulated transforming growth factor (TGF)-β1 in primary cultures of normal human bronchial epithelial cells (NHBE), and RANTES in small airway epithelial cells (SAEC) (Dakhama et al., 2003), which would act *in vivo* together with increased IL-8 production on bronchial epithelial cells (Sohn et al., 2005).

***Alveolar macrophages***. First, Mp attaches to the bronchial epithelial cells. Next, macrophages, including AMs, would play a role as an innate host defense mechanism; however, to our knowledge there are no reports regarding the number of macrophages recruited or pre-existing in the bronchial lumen. AMs are the predominant macrophage type in the lung, constituting approximately 93% of the pulmonary macrophage population (Marriott and Dockrell, 2007). AMs originate from monocytes recruited from the blood, but replication of AMs makes a minor contribution to the total pool (Blusse Van Oud Alblas et al., 1981). In Mp pneumonia, it has been reported that TLR-2 signaling is involved in inflammatory cell activation by Mp-derived lipoproteins (Shimizu et al., 2008). Chu et al. demonstrated that expression of TLR-2 mRNA and protein on alveolar macrophages and the recruitment of adaptor protein MyD88 increase after Mp infection (Chu et al., 2005). AMs are early effectors of innate immunity against any bacteria, and Mp was recognized via TLR1, 2, and 6 on AMs. Previously, studies using our models of germ-free (Hayakawa et al., 2002) and other gnotobiotic mice (Sekine et al., 2009), as well as another study by Chu et al. using BALB/c and C57BL/6 mice (Chu et al., 2006), in turn demonstrated that pre-immunization with live Mp or Mp antigen significantly augmented inflammatory responses after the second challenge. Likewise, Saraya et al. showed enhanced expression of TLR-2 on bronchial epithelial cells and AMs after two immunizations with Mp antigens plus adjuvant alum (Figures 1E, 2E,F) (Saraya et al., 2011; Saraya, 2013). Based on those animal model studies, it is likely that subclinical, latent infection with Mp in the lower respiratory tracts may up-regulate TLR-2 expression on AMs and bronchial epithelial cells, which augments Mp reactivity.

**Figure 6:**
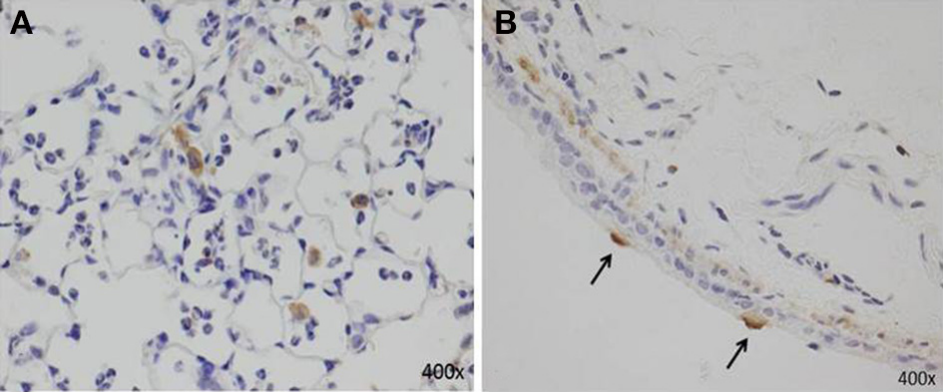
**Immunohistochemical staining with RANTES at 8 h and 48 h post-IT challenge. (A)** AMs secreted RANTES as early as 8 h after IT challenge; abundant recruited neutrophils in alveolar spaces did not produce RANTES. **(B)** Bronchial epithelial cells positive for RANTES at 48 h post-IT.

AMs can also secrete proinflammatory cytokines (IL-6, TNF-α, and IL-1β), IL-18, MIP-1α, KC, RANTES, IL-12, IL-23, and MCP-1 (Saraya et al., 2011; Kurai et al., 2013a; Narita et al., 2000), which are associated with neutrophilic infiltration. Although the number of AMs after two immunizations (models D and E, Figures 1D,E) was equal, we demonstrated that the accumulation of abundant neutrophils in the alveolar spaces as early as 8 h post-IT in model E (Figure 1E) was attributable to the effect of antecedent immunization with Mp antigen, as compared with model D animals (Figure 1D) (Saraya et al., 2011). Vigorous recruitment of neutrophils is one of the most important components of the initial innate immune response (Craig et al., 2009). Immunohistochemical analysis at 8 h post-IT of Model E (Figure 1E) showed that AMs secreted RANTES, which is a known, potent chemoattractant for neutrophils or lymphocytes. However, abundant recruited neutrophils in the alveolar spaces did not produce RANTES (Figure 6A). Bronchial epithelial cells were also immunohistochemically stained with RANTES at 48 h post-IT (Figure 6B).

In this regard, our mouse models for Mp pneumonia (Figure 1E) indicated the possibility that even in humans, latent respiratory infection might trigger the inflammation or enhance the host defense through up-regulation of TLR-2 expression on bronchial epithelial cells and AMs, followed by production of IL-23-dependent IL-17 production (Wu et al., 2007; Kurai et al., 2013a) or other chemokines, including RANTES.

***Lymphocytes***. As mentioned above in the Section “Host defenses,” for human Mp pneumonia, to our knowledge, no data are available regarding whether the presence of lymphocytes in the lung or BALF is due to a specific reaction to Mp. Regarding memory T cells, no combination of chemokine receptors and/or adhesion molecules has apparently been identified to date that imparts a preferential migration to the bronchial compartment or alveolar compartment (Pabst and Tschernig, 1995; Wardlaw et al., 2005; Kohlmeier and Woodland, 2006). Lymphocytes constitute about 10% of all cells in the BALF of healthy adults. Less than 10% of the lymphocytes in the BALF are B cells, and among the T cells, CD4+ cells outnumber CD8+ cells (Pabst and Tschernig, 1997), with a CD4+/CD8+ ratio of 1.7 (Pabst and Tschernig, 1995). There are more so-called “memory” (>85%) than “naive” T lymphocytes in the BALF, which is different from the composition of lymphocytes in other lung compartments (Pabst and Tschernig, 1997). Studies using our mouse model E (Saraya et al., 2011) showed that CCL5 (also known as RANTES) was highly expressed in lung cells, including bronchial epithelial cells, AMs, and lymphocytes. RANTES is produced by activated T cells, fibroblasts, platelets, kidney epithelial cells, macrophages, and endothelial cells, and is chemotactic for memory T cells, monocytes, and eosinophils (Schall et al., 1990; Alam et al., 1993; Monti et al., 1996) as well as neutrophils (Pan et al., 2000), triggering its receptor, CCR5 (Charo and Ransohoff, 2006). Use of our model E demonstrated CCR5-positive lymphocytes in the PBVA, implicating the contribution of RANTES in lung inflammation. Thus, as mentioned in the “Host defenses” Section above, various proinflammatory cytokines and C-C chemokines (RANTES, MCP-1) (Gunn et al., 1997; Johnston et al., 1999) might be key players in the development of Mp pneumonia, both in the acute and chronic phases (Conti and Digioacchino, 2001). Of note, lung pathology seemed to differ according to host characteristics (Th1, Th2, and Th17) which might be a non-specific reaction to Mp.

## Clinical features

### General aspects

Mp infection is usually self-limited and rarely fatal. Mp infection causes both upper and lower respiratory infections, and pneumonia occurs in 3–13% of infected persons (Clyde, 1993). Clinical features of Mp infection vary among different ages, in that patients under 2 years of age tend to have upper respiratory infections, while 6-19-year-olds tend to have pneumonia (Foy et al., 1966; Denny et al., 1971). Two major subtypes of the *P1* gene are known to occur in Mp, and this subtype shift phenomenon may have a relation to Mp pneumonia outbreaks (Kenri et al., 2008). The severity of Mp pneumonia seems to depend on the Mp bacterial load rather than Mp subtype (Nilsson et al., 2010). The incubation period for Mp infection is about 2–4 weeks, and characteristic findings of adult Mp pneumonia are younger age, fewer comorbid diseases, shorter length of stay in hospital, and lower mortality than any other group of CAP patients. Prospective studies of patients with Mp pneumonia from Germany (Von Baum et al., 2009) and Japan (Goto, 2011) revealed average (mean ± SD) ages of 41 ± 16 and 37.7 ± 16.6, respectively.

Severity scores are widely used for assessing the requirement for admission or when describing mortality rates, including the pneumonia severity index (PSI) or CURB-65 (Cilloniz et al., 2011). Gradual onset of respiratory or constitutional symptoms such as cough, fever, headache, and malaise are relatively common symptoms in Mp pneumonia. In particular, dry cough was usually observed in patients during early-phase Mp pneumonia, but it persists for a long period as a typical symptom. Goto (2011) reported that the mean body temperature in adult Japanese patients with Mp pneumonia was 37.7 ± 1.0°C and that 29.2% of patients had a temperature no greater than 37.0°C. Analysis of physical examination data revealed that more than half of patients with Mp pneumonia had no audible crackles and were likely to have late-inspiratory crackles as compared with those infected with typical pathogens (Norisue et al., 2008). On laboratory examination, Mp pneumonia patients had relatively lower leukocyte counts than did those having pneumonia from other causes (Von Baum et al., 2009).

Macrolide was not the preferable treatment for *S. pneumoniae* pneumonia, as opposed to pneumonia from atypical pathogens, including Mp because highly macrolide-resistant *Streptococcus pneumoniae* was emerging to become dominant in Japan (Goto et al., 2009). The Japanese Respiratory Society (JRS) recommended discrimination of atypical pneumonias from CAP due to other pathogens (Committee For The Japanese Respiratory Society Guidelines For The Management Of Respiratory, 2006), and proposed six characteristic signs and symptoms of Mp pneumonia that can easily discriminate the two. Indeed, Yin et al. confirmed that use of these criteria has high sensitivity (88.7%) and specificity (77.5%) (Yin et al., 2012) for the diagnosis of Mp pneumonia if four or more of the proposed factors are present. The six factors are as follows: (i) <60 years of age; (ii) absence of, or only minor, underlying diseases; (iii) stubborn cough; (iv) adverse findings on chest auscultation; (v) absence of sputum or identifiable etiological agent by rapid diagnostic testing; and (vi) a peripheral white blood cell count <10,000/μL.

### Special circumstances

#### Latent respiratory infection/asymptomatic carrier

Mp pneumonia is a one of the leading causes of CAP, and it may exacerbate symptoms of underlying asthma (Nisar et al., 2007), especially in up to 25% of children with wheezing (Henderson et al., 1979); it was identified in 20% of exacerbations in asthmatic children requiring hospitalization and in 50% of children experiencing their first asthmatic attack (Biscardi et al., 2004). Spuesens et al. demonstrated that Mp was carried at high rates in the upper respiratory tracts of healthy children (Spuesens et al., 2013). However, Cunningham et al. could not confirm the relationship between asthma symptoms and Mp infection in children aged 9–11 years (Cunningham et al., 1998). Another study showed that most Mp patients, positive by PCR, had respiratory symptoms; that Mp DNA might be detected several months after acute infection; and that asymptomatic carriage of Mp is uncommon even after the outbreak period (Nilsson et al., 2008).

**Figure 7:**
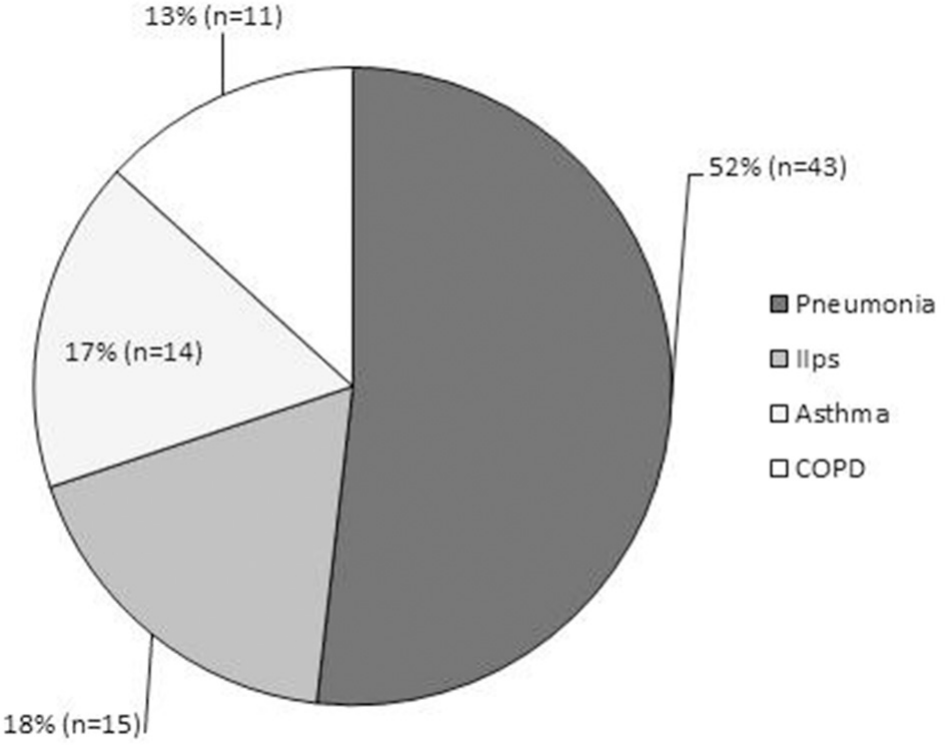
**Epidemiological data of adult patients admitted throughout the year to Kyorin University Hospital for respiratory disease**. Diagnoses consisted of acute exacerbation of idiopathic interstitial pneumonias (*n* = 15, 18%); pneumonia (*n* = 43, 52%), including 4 cases of definite Mp pneumonia diagnosed by CF antibody titer of ≥ four-fold or passive particle agglutination test ≥640; asthma attack (*n* = 14, 17%); and exacerbation of COPD (*n* = 11, 13%).

Especially for adults, to our knowledge, there have been few reports regarding the frequency of latent respiratory infection with Mp. Wadowsky et al. reported that tests of 473 respiratory specimens by culture, PCR, or both identified only four episodes (0.8%) of Mp-associated illness in adolescents and adults (*n* = 491) with persistent cough (Wadowsky et al., 2002). Thus, the frequency of the Mp carrier state or the bacterial load might be different between children and adults, or between healthy and asthmatic individuals. Indeed, our epidemiological data throughout the year demonstrated that among admitted adult patients with diverse respiratory diseases, including acute exacerbation of idiopathic interstitial pneumonia (*n* = 15), pneumonia (*n* = 43), asthma attack (*n* = 14), and exacerbation of COPD (*n* = 11), there were 4 cases of definite Mp pneumonia as diagnosed by a CF antibody titer increased ≥ 4-fold or passive particle agglutination test ≥ 640, but with no identifiable Mp in the throat/nasopharynx or sputum by both culture and PCR methods (Kurai et al., 2013b) (Figure 7). This might reflect the fact that Mp acted only to trigger the lower respiratory symptoms or pneumonia, but the bacterial load was low, resulting in a latent respiratory infection or even in Mp pneumonia, especially in adult patients.

#### Macrolide-resistant Mp pneumonia

Macrolide-resistant Mp emerged and was widespread in East Asia after 2000. The reasons for the regional differences in macrolide-resistant Mp have not been elucidated. The A2063G mutation has been found to be most prevalent in macrolide resistant Mp isolates, followed by the A2064G mutation; these mutations are associated with increased minimum inhibitory concentrations to macrolides, including erythromycin, azithromycin, and clarithromycin.

Previous studies revealed that macrolide-resistant Mp pneumonia patients had a prolonged fever compared to those with macrolide-susceptible Mp pneumonia, in both children and adults (Suzuki et al., 2006; Cao et al., 2010; Pereyre et al., 2012; Yoo et al., 2012). In patients with macrolide-resistant Mp pneumonia, clinical findings, including symptoms, laboratory results, radiology, the complication of respiratory failure, and mortality were not different from those of patients with macrolide-susceptible Mp pneumonia. However, persistent fever over 48 h after initiation of macrolide may point to the presence of macrolide-resistant Mp (Miyashita et al., 2013).

#### Fulminant Mp pneumonia

Mp pneumonia is usually mild and rarely fatal. The severity of Mp pneumonia seems to depend on the Mp bacterial load rather than the Mp genotype (Nilsson et al., 2010). Among patients with Mp pneumonia, 3–4% develop severe, life-threatening illness with respiratory failure and acute respiratory distress syndrome (Holt et al., 1977; Fraley et al., 1979; Koletsky and Weinstein, 1980; Chan and Welsh, 1995; Ito et al., 1995; Takiguchi et al., 2001; Tsuruta et al., 2002; Miyashita et al., 2007). Two groups (Chan and Welsh, 1995; Miyashita et al., 2007) reported that the delayed administration of adequate antimicrobials was noted in severe Mp pneumonia patients, at an average of 9.3 or 15 days, respectively, which may be the most important reason for the development of fatal respiratory failure. However, some cases who had adequate antimicrobials within 3 days after the onset of the disease progressed to respiratory failure (Miyashita et al., 2007). Izumikawa et al. reviewed 52 Japanese cases of fulminant Mp pneumonia (Izumikawa et al., 2014), which was defined as the presence of Mp pneumonia with hypoxia, and reported that no apparent risk factors for fulminant Mp pneumonia were identified, but concluded that initial inappropriate use of antimicrobials may be a risk factor.

## Radiological features

**Figure 8:**
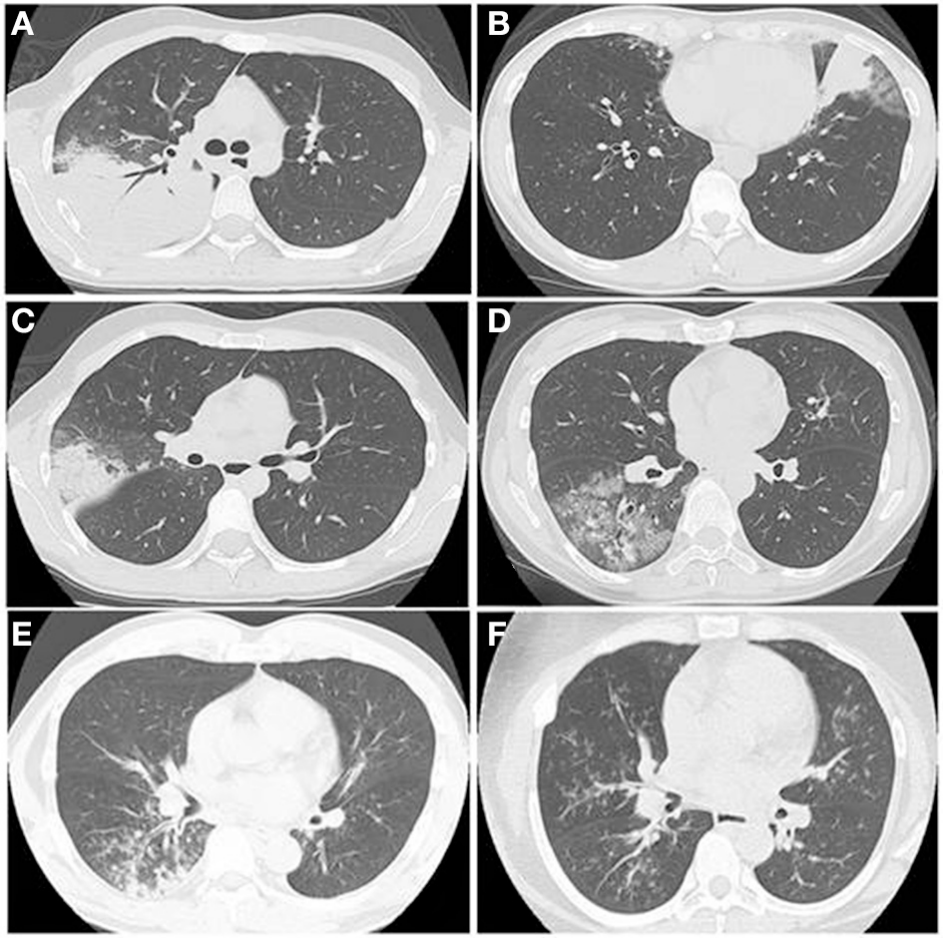
**The HRCT findings of Mp pneumonia are characterized as (A) consolidation with air bronchogram surrounded by a crazy paving appearance, (B) consolidation with reticular shadow, (C) consolidation with GGO, (D) GGO with interlobular septal thickening, crazy paving appearance, (E) bronchial wall thickening with centrilobular nodules, (F) diffuse centrilobular nodules**.

A wide spectrum of findings on thin-section CT have been reported, such as ground glass opacities (GGO), consolidation, bronchial wall thickening, centrilobular nodules, interlobular septal thickening, pleural effusion, mosaic attenuation, air trapping, and lymphadenopathy (Kim et al., 2000; Reittner et al., 2000; Chiu et al., 2006; Lee et al., 2006; Miyashita et al., 2009). Each of those radiological findings are non-specific, but Miyashita et al. reported that bronchial wall thickening and centrilobular nodules on thoracic CT would be a clue to the diagnosis (Miyashita et al., 2009). Figure 8 shows typical HRCT findings such as consolidation with air bronchograms surrounded by a crazy paving appearance (A), consolidation with reticular shadow (B), consolidation with GGO (C), GGO with interlobular septal thickening (D), crazy paving appearance (E), bronchial wall thickening with centrilobular nodules (F), diffuse centrilobular nodules.

## Diagnostic methods

### Culture

Culture is the “gold standard” method for diagnosis of Mp infection and is essential for further analysis, including drug resistance tests, although it is useless as a rapid diagnostic method because of the low sensitivity and the need for incubation for several weeks in specialized culture medium.

### Serological methods

There are many diagnostic serological tests, although these serological tests and their interpretations are not standardized. Serological methods, such as complement fixation (CF), passive agglutination (PA), and detection of IgG and IgM by enzyme immunoassays (EIA) were conventionally used for diagnosis of Mp infection. CF tests measure IgM and IgG antibodies together, but these antibodies are non-specific. The target for PA tests was mainly IgM antibody, but seemed to be less specific for Mp than the Mp-specific IgM enzyme-linked immunosorbent assays (ELISA) (Barker et al., 1990).

Paired sera for CF, PA and Mp-specific IgG EIA tests are widely used for epidemiological studies and are regarded as a standard method for diagnosis. The definition of Mp infection was based on the serological finding of a four-fold titer rise (in CF or PA tests), and seroconversion or a significant increase, of Mp IgG during the convalescent phase compared with the acute phase. Single high titers were also considered markers of Mp infection, and the difference of cut-off titer used in various studies has a great impact on the resulting epidemiological data. If either CF titers are higher than 1:64 or 1:128, or PA titers are higher than 1:320 or 1:640, a diagnosis of Mp infection was made (Marston et al., 1997; Dorigo-Zetsma et al., 1999; Templeton et al., 2003; Beersma et al., 2005; Kim et al., 2007). Measurement of Mp-specific IgM antibodies by EIA has been commercially available for the diagnosis of Mp infection during the early phase. Beersma et al. (2005) reported that twelve IgM EIA assays showed various diagnostic yields when compared to PCR-proved Mp pneumonia as the reference standard. The sensitivity and specificity of these IgM EIA assays were 35–77% and 49–100%, respectively, and those assays had low diagnostic yields within a week after initial onset. Mp-specific IgM (EIA) assays were less useful for adults with autoantibodies or other infectious diseases, such as Epstein-Barr virus, *Streptococcus pyogenes* and *Treponema pallidum*, because of the tendency of these to produce false positives (Petitjean et al., 2002; Beersma et al., 2005).

### Nucleic acid amplification methods

Polymerase chain reaction (PCR)-based methods using respiratory samples have been developed for rapid Mp diagnosis. This application was limited to select hospitals because complicated procedures and expensive systems are required. Diagnosis of Mp infection using PCR was inconsistent among individual studies because of many factors, as follows: patients' ages; intervals between onset of symptoms and sampling specimens; types of specimen sampling methods; target lesion of PCR; and technical procedures (Raty et al., 2005; Loens et al., 2009; Thurman et al., 2009). He et al. showed that PCR-based diagnosis was superior to IgM-based diagnosis in Mp-infected patients less than 3 years of age; an immature immune response to Mp may explain this discrepancy (He et al., 2013). A meta-analysis of PCR-based diagnosis for Mp infection showed that sensitivity and specificity were 0.62 (95% CI, 0.45–0.76) and 0.96 (95% CI, 0.93–0.98), respectively (Zhang et al., 2011).

As for Mp pneumonia, PCR and serological diagnosis had good concordance in adult patients; PCR-based diagnosis had lower sensitivity (66.7%) compared to serological diagnosis as the reference standard. This result was consistent with those in other reports on Mp CAP in adults (Pitcher et al., 2006; Martinez et al., 2008; Qu et al., 2013). The sensitivity and specificity of PCR-based diagnosis in these studies were 40.7–66.7% and 88.8–98.5%, respectively; the reference standard was a serological diagnosis (Table 3).

**Table 3:** **Comparison of diagnostic methods**.

	**Sensitivity (%)**	**Specificity (%)**	**Comment**
Culture	55.6	94.9	Isolation of Mp is slow and insensitive, and therefore is not recommended for routine use.
PCR	40.7–66.7	88.8–98.5	Rapid diagnosis is possible, but is costly and complicated procedures are needed. Therefore PCR-based diagnosis is limited to a few laboratories.
Serology IgM	7.4–77	49–100	Diagnostic yields for Mp IgM tests were variable, according to available assays. Use of paired sera for CF, PA or IgG analysis is preferable.

(Pitcher et al., 2006; Martinez et al., 2008; Qu et al., 2013). Most of data are from lower respiratory infections in adults, including pneumonia patients.

Loens et al. and Raty et al. described that if a sputum sample is available, it might be better for Mp detection in patients with Mp pneumonia than nasopharyngeal or oropharyngeal swabs (Raty et al., 2005; Loens et al., 2009). A nucleic acid amplification method, termed loop-mediated isothermal amplification (LAMP), was introduced in order to improve the complicated system of PCR, and LAMP results were concordant with PCR results (Saito et al., 2005).

In the early phase of the illness, the preferred diagnostic methods seemed to be culture and nucleic acid amplification. In the late phase, those methods are useless because of the low Mp load in the airways; furthermore, regarding the limited value of single serum samples, paired serological examinations would be required for diagnosis (Thurman et al., 2009). In conclusion, no reliable simple method exists for accurate diagnosis; therefore, we recommend the culture and nucleic acid amplification in the early phase, and serological examinations in the late phase, respectively, especially in the patients with severe pneumonia and/or who satisfied four or more of the proposed factors as described in “General aspects.”

## Extrapulmonary manifestations

Although direct invasion, neurotoxin production, or an immune-mediated process have been proposed, the mechanisms underlying extrapulmonary manifestations of Mp infection remain largely unknown. These are diverse (Foy et al., 1983; Lind, 1983; Narita, 2010) and include central nervous system diseases such as encephalitis, aseptic meningitis, polyradiculitis, cerebellar ataxia, and myelitis (Guleria et al., 2005; Tsiodras et al., 2006); cardiovascular diseases such as pericarditis, endocarditis, and myocarditis; the dermatological diseases Stevens-Johnson syndrome, erythema multiforme (Cherry, 1993; Lamoreux et al., 2006), erythema nodosum, anaphylactoid purpura, and acute urticaria (Kano et al., 2007); hematological diseases including autoimmune hemolytic anemia (cold agglutinin disease), hemophagocytic syndrome, disseminated intravascular coagulation, and thrombocytopenic purpura (Cassell and Cole, 1981); inflammatory diseases including conjunctivitis, iritis (Salzman et al., 1992), uveitis (Weinstein et al., 2006), and arthritis (Franz et al., 1997); and otitis media. The presence of these extrapulmonary manifestations is itself evidence of human immune system interaction with Mp.

## Treatment

The recommended therapy for microbiologically confirmed Mp pneumonia is use of macrolides (CAM: clarithromycin and AZM: azithromycin) or tetracyclines, and fluoroquinolones are an alternative choice (Lim et al., 2009). However, neither tetracyclines nor fluoroquinolones are recommended for young children under 8 years of age because of their adverse effects, such as permanent yellowing or graying of the teeth, and abnormalities of articular cartilage and the QT interval. Therefore, macrolide-resistant Mp pneumonia is a major concern for children who require treatment. Several studies showed that macrolide-resistant Mp was susceptible to tetracycline and fluoroquinolone *in vitro* (Eshaghi et al., 2013; Hong et al., 2013). Minocycline or doxycycline, both tetracyclines, quickly decreased the loads of macrolide-resistant Mp and were effective against the resistant pathogen in humans. Okada et al. showed that tosufloxacin, a fluoroquinolone, seemed to be inferior to minocycline or doxycycline in clinical use (Okada et al., 2012). However, macrolides have an immunomodulatory or bacteriological effects even on a mouse model with macrolide-resistant Mp strain (Kurata et al., 2010). Therefore, even in the area of high resistant to macrolides such as Japan, JRS recommend the use of macrolides as first therapy for Mp pneumonia together with the use of method for differential diagnosis of atypical pneumonia and bacterial pneumonia.

Infectious Diseases Society of America (IDSA) and the American Thoracic Society (ATS) joint guidelines on adult CAP described that patients with CAP should be treated for a minimum of 5 days (level I evidence), and most patient become clinically stable within 3–7 days, so longer durations of therapy are rarely necessary (Mandell et al., 2007), but JRS guidelines does not refer to the optimal duration of the treatment. Smith CB et al. showed that tetracycline and erythromycin improve symptoms in adult volunteers who experimentally infected with Mp, but recurrence of Mp pneumonia was noted after completion of 7 days treatment with tetracycline (Smith et al., 1967).

Thus, the optimal antimicrobial dosage and duration are not clear; however, 10–14 days of therapy is generally recommended. Effective treatment of Mp pneumonia shortens the duration of fever and might prevent aggravation (Denny et al., 1971; Izumikawa et al., 2014).

### Immunomodulative effects of macrolide therapy

Macrolides have direct effects on neutrophil function and production of cytokines involved in inflammation cascades (Zarogoulidis et al., 2012).

For Mp infections, 14- or 15-membered ring macrolides usually are considered the first-line agents, which are well known for anti-inflammatory, immunomodulative effects (Wales and Woodhead, 1999). CAM is a macrolide with a 14-atom lactone ring, and attenuation of inflammatory responses has been reported in both animal models of Mp pneumonia (Kurata et al., 2010) and in humans with respiratory diseases (Kudoh et al., 1998).

**Figure 9:**
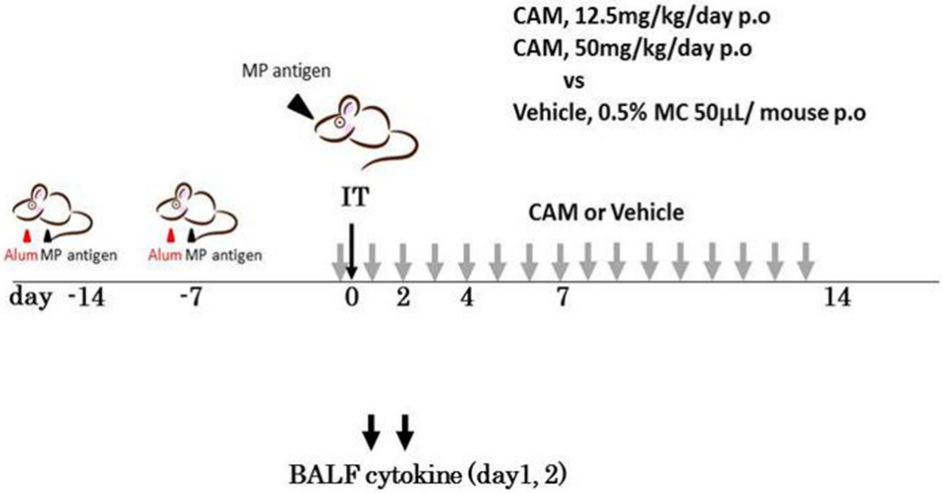
**Model E, the clarithromycin (CAM)-treated murine model**. Just before intratracheal challenge (IT) with Mp antigen, mice were divided into 3 treatment regimen groups. All were orally treated once a day with CAM (12.5 mg/kg/day or 25 mg/kg/day) or vehicle for 2 consecutive weeks. CAM, clarithromycin; IT, intratracheal challenge; P.O, per os; BALF, bronchial alveolar lavage fluid.

**Figure 10:**
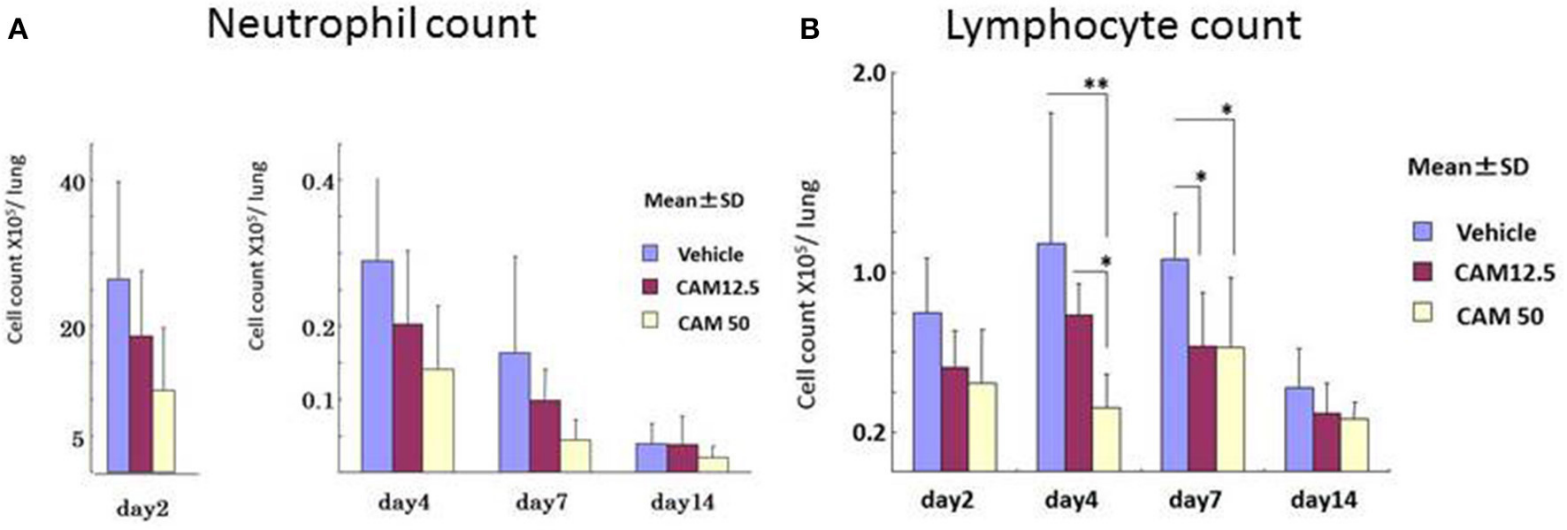
**Sequential analysis of bronchial alveolar lavage fluid at days 2, 4, 7, and 14 after intratracheal challenge. (A)** No significant difference in the neutrophil count was seen. **(B)** Number of lymphocytes in the CAM-treated groups was significantly suppressed dose-dependently at days 4 and 7.

**Figure 11:**
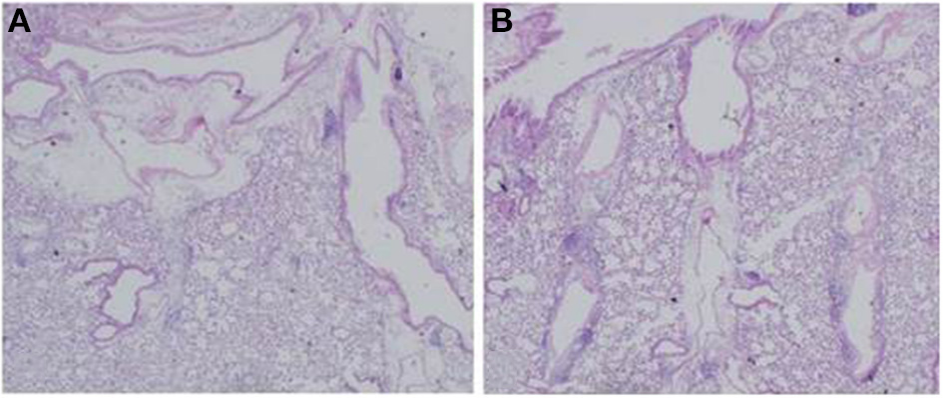
**Lymphoplasmacytic infiltration within the PBVA was markedly suppressed in the (A) CAM50 group as compared with the (B) vehicle group. PBVA, peribronchovascular area**.

To examine the immunomodulative effects of CAM, mice in model E (Figure 1E) were treated with three different regimens, as follows: (Figure 9) orally with CAM at two doses (CAM12.5 group: 12.5 mg/kg/day or CAM50 group: 50 mg/kg/day); or with vehicle (methylcellulose), all at 1.5 h just before IT with Mp antigen (day 0) (Saraya et al., 2007a; Saraya and Goto, 2008). Just before and after IT, the 3 groups were orally treated once a day with CAM or vehicle for 2 consecutive weeks. On BAL cell differential count analysis, there were no significant differences in the neutrophil count among the 3 groups in any phase (Figure 10A). However, the number of lymphocytes in the CAM-treated groups was significantly suppressed in a dose-dependent manner at day 4, and the effect was still recognized at day 7 (Figure 10B). Pathological assessment at day 4 post-IT revealed that the lymphoplasmacytic infiltration within the PBVA was markedly suppressed in the CAM50 group (Figure 11A), as compared with that of the vehicle group (Figure 11B).

**Figure 12:**
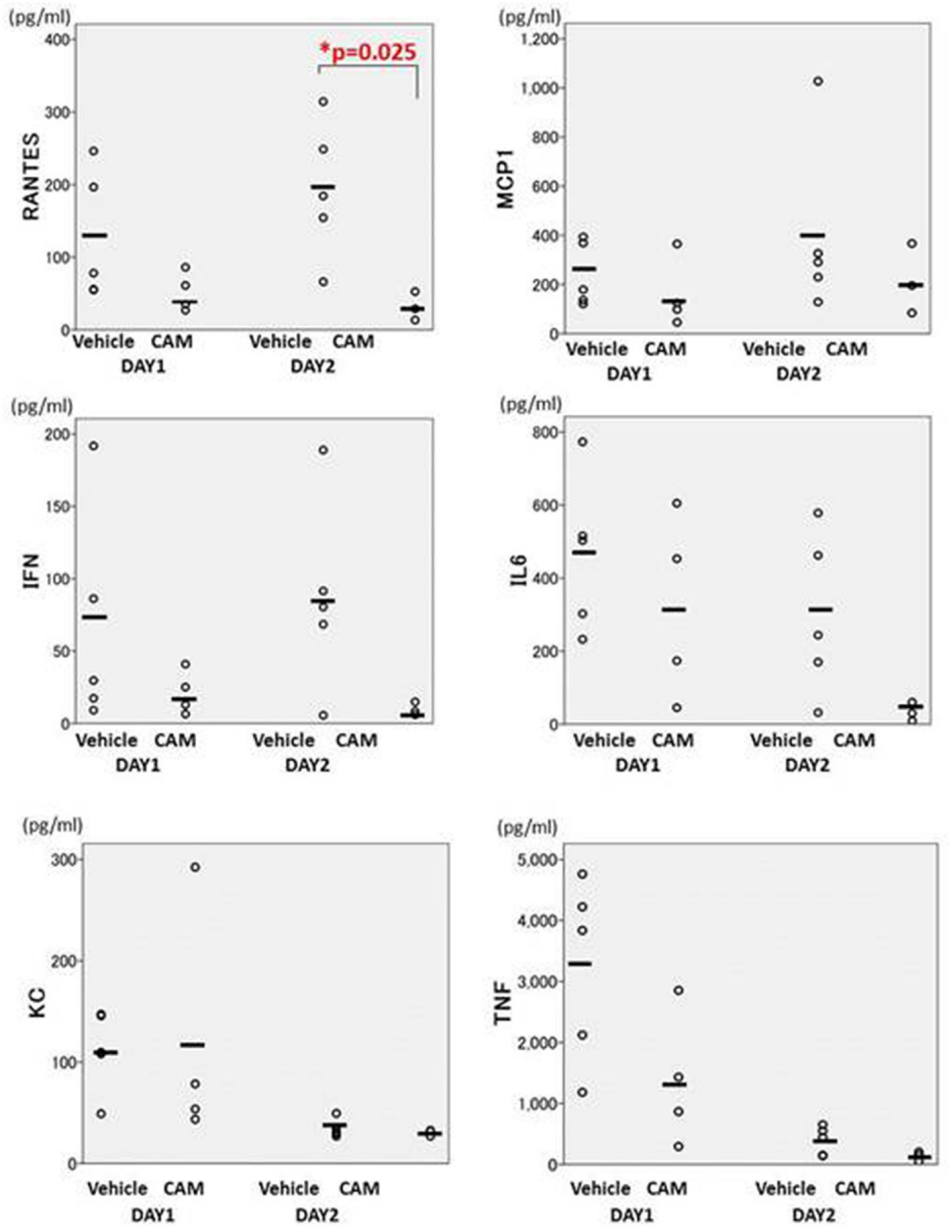
**There were fewer cytokines in the CAM50-treated group (50 mg/kg/day orally), compared with the vehicle group**. Only RANTES was significantly suppressed in the CAM50 group, at day 2 (*p* = 0.025). BALF: bronchoalveolar lavage fluid. RANTES: regulated on activation, normal T cell expressed and secreted. ^*^p < 0.05.

BALF cytokines in the CAM50 group seemed to be lower than those of the vehicle group, and only RANTES was significantly suppressed in the former group, at day 2 (*p* = 0.025) (Figure 12). Those data suggested that oral administration of CAM has immunomodulative effects on lung inflammation even in the early phase of Mp pneumonia. This dose-dependent immunomodulative effect of CAM was consistent with previously reported results of a study using an experimental Mp pneumonia mouse model (Tagliabue et al., 2011).

### Steroids as additive therapy

Animal experimental models (Tagliabue et al., 2008; Hirao et al., 2011) showed that corticosteroids down-regulate the host immune response. Furthermore, treatment with the combination of clarithromycin and a corticosteroid, compared to clarithromycin alone, resulted in a significantly greater reduction of IL-12 p40 and RANTES (Tagliabue et al., 2008). Izumikawa et al. (2014) reported that a majority of human patients with fulminant Mp pneumonia had improved respiratory function on steroid treatment within 3–5 days, which was considered to be an effect of suppressing hyperactivated cellular immunity. Radisic et al. reported on the suppressing effects of steroids on the cell-mediated immune response (Radisic et al., 2000), and that acute respiratory distress syndrome (ARDS) secondary to Mp infection is a lymphoid cellularity ARDS caused by a harmful, “over-reacting” cell-mediated immune response, which could potentially be tapered by the use of steroids. Thus, steroid use would be the preferable treatment of patients with fulminant Mp pneumonia in light of the immune response.

### Conflict of interest statement

The authors declare that the research was conducted in the absence of any commercial or financial relationships that could be construed as a potential conflict of interest.
